# Driven by feelings or stimulated by context: how childhood nature experience shaped adulthood pro-environmental behavior?

**DOI:** 10.3389/fpsyg.2025.1529388

**Published:** 2025-03-03

**Authors:** Qi-song Yan, Yu-fang Cai, Wen-qiang Zeng

**Affiliations:** ^1^School of Management, Chongqing University of Science and Technology, Chongqing, China; ^2^Personnel Education Department of Yunnan Academy of Social Sciences, Kunming, China; ^3^School of Sociology, Yunnan Minzu University, Kunming, China

**Keywords:** childhood nature experiences, environmental feelings, environmental contacts, pro-environmental behaviors, environmental risk perception

## Abstract

**Introduction:**

In recent years, China has vigorously promoted sustainable social development, aiming to enhance residents’ environmental protection awareness and encourage their active participation in environmental protection through various means. To achieve this goal, cultivating environmental feelings (EF) among residents has become a key initiative. Childhood nature experiences (CNE) may have a profound impact on pro-environmental behaviors (PEB) in adulthood. However, the specific mechanisms underlying this influence remain unclear.

**Methods:**

This study, viewed through the lens of the biophilia hypothesis, uses EF and environmental contacts (EC) as mediating variables, and environmental risk perception (ERP) as a moderating variable. Statistical analyses, including multiple linear regression, mediation, and moderation analyses, were conducted on data from 1,499 survey responses to explore the mechanisms through which CNE influence PEB in adulthood.

**Results:**

(1) The study shows that CNE do not have a direct effect on PEB in adulthood, but instead exert an indirect influence through EF and EC, with the mediating effect of EF being stronger than that of EC. (2) ERP significantly moderates the relationship between EC and two types of PEB, as well as the link between EF and private sphere pro-environmental behaviors (PRIEB). However, it does not significantly moderate the relationship between EF and public sphere pro-environmental behaviors (PUBEB). (3) ERP significantly moderates most of the mediating effects.

**Conclusion:**

The findings suggest that relying solely on childhood EC does not directly shape adult PEB. Compared to EC, EF play a larger mediating role between CNE and adult PEB. ERP strengthens the mediating effects of both EC and EF. The study emphasizes that both unstructured nature contact and planned, educational outdoor activities during childhood are equally important. Society should provide abundant opportunities for nature experiences, cultivate environmental feelings, and establish a close connection with nature to lay the foundation for developing future participants and advocates for environmental protection.

## 1 Introduction

After more than four decades of modernization, the residents of Chinese society have raised higher expectations for the quality of the ecological environment. To address this, the Chinese government has implemented a series of measures over the past 10 years to advance the construction of ecological civilization, achieving significant results. In this process, the Chinese government has particularly emphasized the involvement of residents in ecological civilization construction and actively taken measures to promote the active participation of various sectors of society. The Chinese government has worked on popularizing environmental knowledge among residents to enhance their environmental awareness, action capacity, and the sustainable development of ecological civilization. The methods and effectiveness of environmental knowledge dissemination vary for different groups. Previous studies have shown that the environmental attitudes formed in childhood tend to be stable, and the earlier a child forms a positive environmental attitude, the less likely it is to be changed (Braun and Dierkes, 2017). Therefore, children are considered the ideal target for environmental knowledge dissemination. Children who receive environmental education are more likely to adopt PEB, which also helps cultivate their long-term environmental behavior (Iwasaki, 2022). Effectively enhancing children’s environmental awareness and promoting the formation of their PEB have always been hot topics in environmental protection. The dissemination of environmental knowledge is regarded as an effective method to promote PEB, as it emphasizes systematically teaching environmental knowledge to increase rational awareness of the ecological environment, raise attention to environmental issues, and foster the ability to analyze environmental problems (Otto and Pensini, 2017; Ardoin et al., 2020; Van De Wetering et al., 2022). Theories from rational perspectives, such as the Theory of Planned Behavior and the Value-Belief-Norm (VBN) Theory, are commonly used to explain the formation mechanisms of PEB in adults (Stern and Dietz, 1994). However, in practice, residents’ environmental behavior does not solely stem from rational thinking. Emotional factors such as affinity for nature, interest, and anger also play a significant role in analyzing PEB (Kals et al., 1999). Moreover, because children’s cognitive abilities are usually not developed enough to conduct complex rational analysis, explanations of children’s PEB from a rational perspective lack sufficient explanatory power.

Given the limitations of rational perspectives in explaining children’s PEB, theories from an emotional perspective are more persuasive in elucidating the relationship between children’s experiences and PEB. A typical example is the biophilia hypothesis proposed by Wilson. The biophilia hypothesis posits that humans have an inherent connection with the natural environment and its organisms, and through interactions with nature, humans can experience pleasure and tranquility, which in turn strengthens their awareness of care and protection for the natural environment (Wilson, 1984). The biophilia hypothesis suggests that in unconstrained settings, people can derive enjoyment from the natural ecological environment (Rosa and Collado, 2019), especially in certain natural contexts that encourage repeated interaction with nature, fostering emotional connections and a sense of responsibility toward nature and its life forms (Kellert and Wilson, 1993). Outdoor nature experiences are also a form of promoting children’s environmental awareness and behavior. CNE refer to the direct interactions children have with the natural environment during their growth, including outdoor play, observing plants and animals, and participating in nature-based activities (Ardoin and Bowers, 2020; Liu and Green, 2023), through which they gain perceptions of nature and establish EF (Bratman et al., 2015). In nature experiences, children are encouraged to explore freely, engage in activities like field observation and planting, and experience the joy brought by the natural environment, fostering their observation skills, curiosity, and creativity (Miller et al., 2021). By directly interacting with nature and engaging in emotional experiences, children not only witness the beauty of nature firsthand but also deepen their understanding of ecological system functions through observation and exploration activities, thereby forming their initial emotional connections with the natural environment (Cottrell and Cottrell, 2020; Kiviranta et al., 2024). This enhances children’s willingness to engage with nature, effectively stimulating their environmental motivation, environmental capabilities, and PEB (Kahn and Kellert, 2002; Barbiero and Berto, 2021; Sprague et al., 2021).

Previous studies have provided important references for understanding the impact of CNE on PEB. However, there are still limitations in past research. First, most previous studies have only examined the relationship between environmental awareness and PEB during childhood, without clearly addressing whether CNE influence EC or EF in adulthood (Ewert et al., 2005). While CNE can enhance environmental awareness and behavior in adulthood and influence some individuals’ choice of an environmental career (Rosa and Collado, 2019; Cleary et al., 2020), their impact on dealing with environmental risks such as climate change in adulthood is not significant (Howell and Allen, 2016). This indicates that the influence of CNE on PEB in adulthood is not consistent, and further research is needed to clarify these findings. Second, past studies have lacked comparative analyses of the mechanisms by which CNE affect PEB in adulthood. EC and stable, lasting EF in specific contexts are two important dimensions of nature experiences (Clayton et al., 2019). EF refer to a stable, enduring, and implicit attitude that arises when an individual interacts with the natural environment, based on whether their needs are met (Conte et al., 2023). EC refer to any interactions between humans and the flora, fauna, and geological features of the natural environment (Martin et al., 2020; Liu et al., 2022), such as walking in a park, rock climbing, or pruning branches. EC are overt behaviors, often triggered by specific contexts, and are less stable and lasting than EF. EF may drive PEB more effectively than the triggering effect of EC (Lin et al., 2014). However, past research has not delved into how CNE shape EF and EC in adulthood, nor has it clearly compared the relative importance of lasting EF versus context-specific EC in influencing PEB. Third, although previous research has analyzed the relationships between EF, EC, and PEB, there has been insufficient comparative analysis of these relationships in different contexts. While environmental pollution caused by industrialization and urbanization in China has eased, there are still differences in the effectiveness of environmental governance across regions (Yu, 2014; Lan et al., 2016), and residents’ perceptions of environmental risks also vary. Residents’ ERP is a key contextual factor that may moderate the relationship between EF, EC, and PEB. A deeper analysis of its moderating effects will help understand the mechanisms by which CNE influence PEB in adulthood, which is crucial for environmental policy development.

Currently, children in China, especially those living in towns and cities, are constrained by various factors such as academic pressure. They spend more time receiving classroom education and have fewer opportunities for outdoor free play and direct contact with nature. This limited exposure hinders the development of direct emotional experiences with the natural environment in childhood, which in turn may negatively affect the formation of health-conscious and PEB in the future (Ardoin and Bowers, 2020). Against this backdrop, it is increasingly important to encourage children to engage with the natural environment and cultivate EF from a young age to foster better PEB in adulthood. Therefore, in order to more comprehensively understand and promote the societal value of CNE, it is necessary to empirically analyze and clarify the effects and mechanisms of CNE on PEB in adulthood, as well as to compare the effects of EF and EC in promoting PEB in adulthood.

This study, framed within the perspective of the biophilia hypothesis, aims to reveal how childhood natural experiences shape environmental behaviors in adulthood. The core issue focuses on exploring whether it is enduring EF or situational EC that play a more critical role in influencing environmental behaviors in adulthood. Additionally, we will investigate how ERP moderates this mechanism. In the context of China’s active promotion of ecological civilization and sustainable social development, this research extends the explanatory scope of the biophilia hypothesis and provides important insights for the formulation of policies aimed at fostering children’s environmental sentiments and mobilizing residents’ participation in environmental protection.

## 2 Literature review and research hypothesis

### 2.1 Natural experience and pro-environmental behavior

Positive CNE are key factors in shaping PEB. These experiences strengthen the connection between children and nature (Barton et al., 2016; Evans et al., 2018), and CNE can also motivate individuals to engage in behaviors such as participation in environmental policy-making, energy conservation, and resource recycling (Chawla and Cushing, 2007; Chawla, 2021). The influence of CNE on PEB in adulthood occurs mainly through two pathways: value cultivation and EF. First, CNE not only enhance the environmental sensitivity and values of both children and adults, but also cultivate their tendencies toward PEB (Armbruster, 2006). Integrating classroom learning with community environments can stimulate children’s intrinsic environmental concern and nature experiences, which in turn promotes their PEB in adulthood (Chawla, 1998; Cleary et al., 2017). Second, the EF pathway suggests that nature experiences can foster PEB by promoting individuals’ EF and mental health (Wolsko and Lindberg, 2013). Satisfying experiences in nature can enhance individuals’ emotional connection to the environment, thereby strengthening children’s commitment to environmental protection and potentially increasing their likelihood of engaging in PEB (Lee, 2011). Unconstrained nature contact and encouraged natural exploration help children form emotional bonds with the environment and develop environmental identity [37], further cultivating positive and lasting environmental attitudes and a sense of responsibility (Knopp and Tyger, 1973; Ngo et al., 2019). Overall, CNE play a formative role in fostering PEB. Based on this, we propose Hypothesis 1.

*H1*: CNE are positively correlated with PEB in adulthood. The better the CNE, the higher the level of PEB in adulthood.

### 2.2 Childhood natural experience and environmental feelings

Childhood is a critical stage for shaping EF, where children, through direct nature experiences, can form both cognitive and emotional connections to the environment (Giusti et al., 2014). The relationship between CNE and EF is typically explained by two theoretical frameworks: the situational stimulus theory and the need fulfillment theory. Chawla, a prominent figure in the situational stimulus theory, argues that positive experiences in natural environments are crucial for fostering responsible EF. Children exposed to outdoor natural settings can gain richer creative experiences and positive feedback, thereby stimulating deeper environmental awareness and interest, among other positive emotions (Chawla, 1999). Nature play is a key factor in shaping EF, as it stimulates children’s sensory systems, provides rich physical experiences, and deepens their perception and emotional connection to the environment (Lohr and Pearson-Mims, 2005; Chawla, 2021). Both natural and built environments can help children understand environmental degradation and form ecocentric EF (Ewert et al., 2005), though outdoor natural stimuli are more effective in enhancing these EF (Braun and Dierkes, 2017). From the perspective of the need fulfillment theory, children may face social tension during their socialization process. When nature experiences offer comfort and satisfy psychological needs, children are likely to develop positive emotions toward the environment; conversely, negative nature experiences may weaken the emotional connection to the environment (Cleary et al., 2017). Positive experiences in natural environments can promote psychological recovery in children, reduce stress and fatigue, and enhance their willingness to engage in PEB (Ulrich, 1984). In summary, nature experiences play a significant role in shaping children’s feelings connection to the environment. Leads to the formulation of the second hypothesis of this study.

*H2*: CNE have a positive association with EF.

### 2.3 Childhood natural experience and environmental contacts

CNE are closely linked to EC and PEB in adulthood, not only fostering an attachment to the natural environment (Beery and Jørgensen, 2018), but also influencing long-term concern and protective actions toward it. These early experiences stimulate an interest in exploring nature and instill environmental protection awareness, which promotes active participation in environmental activities during adulthood (Chawla, 1999). Nature experiences are considered a crucial prerequisite for individuals to engage with and further understand the natural environment (Chawla, 1998). Outdoor education activities, such as field trips and summer camps, significantly increase opportunities for children to have environmental contacts (Wolsko and Lindberg, 2013; Lin et al., 2014). Children not only have the chance to personally experience the joys of nature but also learn ecological knowledge and survival skills, which help establish a deeper connection with the environment (Williams and Chawla, 2016). The sense of involvement in natural environments is an intrinsic motivator that encourages children to maintain EC (Chawla, 2021; Keith et al., 2022). Nature experiences are a continuous process; both childhood and adulthood nature experiences can influence the likelihood of EC, but CNE have a stronger predictive effect on an individual’s current level of EC (Broom, 2017; Cleary et al., 2020). Additionally, CNE can promote social interactions and community involvement (Roberts et al., 2019; Li et al., 2024), which help reduce stress and improve mood, thereby encouraging further engagement with the natural environment (Martin et al., 2020). Existing research has shown that CNE have a profound and lasting impact on an individual’s long-term EC, leads to the formulation of the third hypothesis in this study.

*H3*: CNE have a positive association with EC.

### 2.4 Environmental feelings, environmental risk perception, and pro-environmental behavior

The relationship between EF and PEB is primarily explained by two theories: emotional activation theory and emotional function theory. Emotional activation theory, inspired by norm activation theory, views emotional feelings as a critical resource for environmental protection. Contact with the natural environment can enhance an individual’s behavioral skills and sense of control, activating a sense of environmental responsibility and behavioral intention (Yan et al., 2024), which significantly promotes PEB (Reis and Roth, 2009). If EF are not effectively activated, they, along with environmental knowledge, will not effectively influence PEB (Gezhi and Xiang, 2022). The stronger the sense of comfort individuals feel when engaging with the natural environment, the more likely their EF will be activated, thus increasing the likelihood of PEB (Weber, 2006; Cleary et al., 2020). Emotional function theory, on the other hand, posits that EF encompass various types such as fear, hope, anger, and interest. EF arise in specific contexts and drive different PEB. When environmental degradation causes emotional distress, individuals will attempt to alleviate negative feelings through PEB (Kasser, 2017). In cases where the relationship between EF and PEB is confirmed, emotional interventions can be appropriately applied to promote the formation of PEB (Dohle et al., 2010; Williamson and Thulin, 2022). However, the relationship between EF and PEB may be disrupted by factors such as the lack of EF. When individuals lack awareness of environmental risks and their consequences, they may not experience EF and consequently lack the motivation to engage in environmental protection activities (Testa et al., 2021). When individuals have a higher perception of environmental risks, their EF may be activated (Latif et al., 2024), which in turn drives them to take action to reduce environmental risks (Zeng et al., 2020; Zhang et al., 2024b). For example, when people perceive lower environmental risks, place attachment can promote PEB (De Dominicis et al., 2015). Risk perception can also strengthen residents’ green identity, indirectly influencing their PEB (Bradley et al., 2020). Based on this, we propose Hypothesis 4.

*H4a*: EF positively promote PEB. The higher an individual’s EF, the higher their level of PEB.

*H4b*: ERP positively moderates the relationship between EF and PEB.

### 2.5 Environmental contacts, environmental risk perception, and pro-environmental behavior

The biophilia hypothesis posits that humans instinctively seek connections with nature, which is not only fundamental for survival and development but also fosters a sense of dependency on nature (Wilson, 1984). The theory of nature connectedness further suggests that individual contact with natural environments can enhance appreciation and respect for nature. Individuals with more frequent contact with nature tend to place greater value on nature’s worth (Cleary et al., 2017). People with a stronger sense of connection to nature are more likely to engage in PEB such as waste sorting (Lin et al., 2014). The longer individuals are exposed to nature, the more likely their natural environment protection behaviors are to be activated (Carmi et al., 2015). Moreover, close interactions with nature not only improve individual mood but also deepen understanding of environmental issues, enhancing willingness to engage in environmental protection. This interaction is also a key driver of social sustainability (Li et al., 2021; Zelenski and Desrochers, 2021). While previous studies have not directly analyzed the moderating role of ERP in the relationship between EC and PEB, some related studies suggest that when individuals perceive environmental risks, they seek to re-establish connections between humans and the natural environment to address these risks (Braun and Dierkes, 2017). Residents’ concerns about air pollution may lead them to regularly check air quality indexes (Adebayo-Ojo et al., 2023). Similarly, individuals with a deep awareness of climate change are more likely to participate in environmental protection organizations and opt for public transportation to reduce energy consumption (Han et al., 2017). ERP can increase individuals’ EC, heighten sensitivity to environmental issues, and promote more active PEB (Stern, 2000). Based on these insights, we propose the fifth hypothesis of this study.

*H5a*: EC positively promote PEB. The more an individual is exposed to EC, the higher their level of PEB.

*H5b*: ERP positively moderates the relationship between EC and PEB.

**Figure 1:**
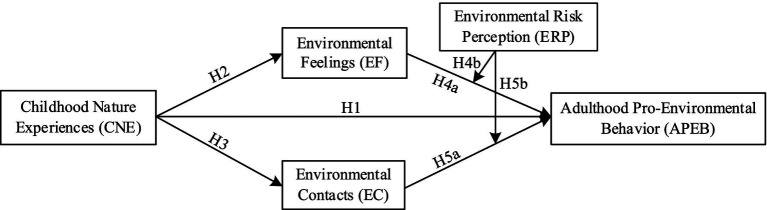
Analysis framework diagram.

As shown in Figure 1, the analytical framework clearly illustrates the relationships among the key variables in this study.

## 3 Method

### 3.1 Participants

The data used in this study were derived from a questionnaire survey conducted in 2020 across nine provinces and cities in China, including Chongqing, Hubei, Shandong, Henan, Shanxi, Sichuan, Fujian, Guangdong, and Guizhou. The survey targeted residents with Chinese nationality or household registration, and multi-stage random sampling was employed to obtain the sample. To refine and improve the questionnaire, the research team consulted experts and conducted a pilot survey. After modifying the questionnaire based on the feedback, the research team recruited university students specializing in social sciences as investigators and provided them with standardized training. The training covered the research topic, understanding of the questionnaire, survey techniques, and sampling methods. During the survey, after obtaining respondents’ consent and clearly explaining the purpose of the survey and the confidentiality of the data, the investigators conducted face-to-face interviews using handheld questionnaires. A total of 1,499 valid questionnaires were collected. The participants exhibited a balanced distribution across key demographic characteristics, including gender, age, educational background, family annual income, and place of residence, ensuring that the sample accurately reflected the overall population structure. A detailed breakdown of the demographic distribution is presented in Table 1. The focus of this study is the relationship between CNE and PEB. The five core concepts—CNE, PEB, EF, EC, and ERP—were all measured using a five-point Likert scale.

**Table 1:** Descriptive statistics of sample distribution (*n* = 1,499).

Variable	Range	Frequency	Percentage (%)
Age	Under 29 years old	503	33.6
	30–44 years old	415	27.7
	45–59 years old	482	32.2
	Over 60 years old	99	6.6
Gender	Female	848	56.57
	Male	651	43.43
Educational level	Primary school and below	178	11.88
	Junior high school	302	20.15
	Senior high school	274	18.28
	Junior college	220	14.68
	Undergraduate	481	32.09
	Postgraduate or above	44	2.94
Annual household income (RMB)	Below 30,000	248	16.5
	30,000–100,000	718	47.9
	100,000–200,000	347	23.1
	More than 200,000	186	12.4
Marriage	Married	919	61.31
	Unmarried	580	38.69
Place of residence	Urban	962	64.18
	Rural	537	35.82

### 3.2 Variable measurement

The dependent variable in this study is PEB. The measurement of PEB is adapted from the scales of Kollmuss and Agyeman (2002) and Markle (2013), with certain modifications. It includes 10 items measuring PRIEB, such as “I separate the waste at home” and “I turn off the lights when I am the last one to leave the room.” PUBEB is measured by 7 items, such as “donating for the environmental protection of the Yangtze and Yellow River basins” and “participating in environmental activities organized by non-governmental environmental groups.” The options for both types of PEB are “none at all,” “less,” “average,” “more,” and “often,” which are coded in order from 1 to 5. The Cronbach’s alpha value for PRIEB is 0.789, and the KMO value is 0.845. The Cronbach’s alpha value for PUBEB is 0.890, and the KMO value is 0.905. Both types of PEB have good reliability and validity. Factor scores are obtained for the two types of PEB through factor analysis. The higher the factor score, the higher the level of corresponding PEB.

The independent variable in this study is CNE, adapted from the Nishet’s Nature Connection Scale (Nisbet and Zelenski, 2011, 2013), with modifications to better suit the Chinese context. The scale primarily includes four questions: “In my childhood, I participated in many interesting outdoor activities,” “I could always find enjoyment in the garden or fields near my home when I was a child,” “I felt very happy when I went to the forest with my parents during my childhood,” and “The streams and fields in my hometown during childhood always brought me joy.” This study codes “completely inconsistent” as 1, “not very consistent” as 2, “average” as 3, “relatively consistent” as 4, and “very consistent” as 5. The Cronbach’s alpha value for the four items is 0.655, and the KMO value is 0.622. Both reliability and validity values are within an acceptable range. A single factor is combined through principal component analysis. The higher the factor score, the better the childhood nature experience.

The mediating variables in this study are EF and EC. These were measured using an adapted version of the Children’s Nature Connection Scale developed by Perkins (2010). EF include six items such as “I am inseparable from nature; I am a part of nature,” and “Wherever I am, I make a conscious effort to pay attention to local wildlife,” to assess EF. The options are “completely disagree,” “disagree,” “unclear,” “somewhat agree,” and “completely agree,” which are coded in order from 1 to 5. The Cronbach’s alpha value for the six items is 0.745, and the KMO value is 0.773. Both reliability and validity values are good. An EF factor is synthesized through the principal component method. The higher the factor score, the higher the level of EF.

EC are measured by 3 items, which are “In the past 6 months, how often have you taken care of plants or flowers at home (watering, loosening the soil, fertilizing, pruning leaves, etc.)?,” “In the past 6 months, how often have you been in contact with the natural environment (visiting parks, visiting green belts in the community, going on outings, climbing mountains, etc.)?,” and “In the past 6 months, how often have you participated in outdoor activities (such as hiking, cycling, camping)?.” The answers are “never,” “1–2 times a month,” “once a week,” “every 2–3 days,” and “almost every day,” which are coded in order from 1 to 5. The Cronbach’s alpha value for the three items is 0.869, and the KMO value is 0.737. Both reliability and validity values are within an acceptable range. An EC factor is synthesized through the principal component method. The larger the factor, the greater the frequency of EC.

In this study, ERP is treated as a moderating variable, with the measurement method adapted from the Chinese General Social Survey (Su et al., 2021). The specific measurement method is as follows: it analyzes the extent to which environmental issues or risks pose a significant threat to us. The questionnaire asks respondents 13 questions, such as “air pollution,” “water pollution,” “noise pollution,” “soil pollution,” “domestic waste pollution,” “lack of green space,” etc. The options are “not applicable,” “not very serious,” “average (unclear),” “quite serious,” and “very serious,” which are coded in order from 1 to 5. The Cronbach’s alpha value for the 13 items is 0.955, and the KMO value is 0.959. Both reliability and validity values are very high. Through the principal component analysis method, the values of the 13 items are combined into an ERP factor. The larger the value, the greater the environmental risk perceived.

The control variables in this study include gender (male = 1, female = 0), age, residence (rural = 0, urban = 1), family annual income, and years of education. Among these, gender and residence are binary variables, while age, family annual income, and years of education are continuous variables.

Table 1 presents the descriptive statistics of the sample distribution in this study. The sample includes 503 individuals (33.6%) under the age of 29, 415 individuals (27.7%) aged between 30 and 44, 482 individuals (32.2%) aged between 45 and 59, and 99 individuals (6.6%) aged 60 and above. Females constitute 56.57% (848 individuals) of the total sample, while males make up 43.43% (651 individuals). In terms of educational attainment, 178 individuals (11.88%) have primary school education or less, 302 individuals (20.15%) have junior high school education, 274 individuals (18.28%) have senior high school education, 220 individuals (14.68%) have associate degree education, 481 individuals (32.09%) have undergraduate education, and 44 individuals (2.94%) have postgraduate education or higher. This indicates that individuals with undergraduate education make up the largest proportion of the sample, followed by those with junior and senior high school education. Regarding annual income, 248 individuals (16.5%) have a family annual income of less than 30,000 RMB, 718 individuals (47.9%) have a family annual income between 30,000 and 100,000 RMB, 347 individuals (23.1%) have a family annual income between 100,000 and 200,000 RMB, and 186 individuals (12.4%) have a family annual income exceeding 200,000 RMB. In terms of marital status, married individuals account for 61.31% (919 individuals) of the total sample, while unmarried individuals account for 38.69% (580 individuals). 962 individuals (64.18%) reside in urban areas, while 537 individuals (35.82%) reside in rural areas. The sample distribution suggests that the population analyzed in this study is diverse, indicating a strong representativeness of the sample.

### 3.3 Statistical analysis methods

We primarily employed Stata 17 statistical software for data analysis. The multiple linear regression model effectively evaluates the strength and direction of the impact of multiple independent variables on the dependent variable. Therefore, a multiple linear regression model is first used to analyze the effects of CNE, EF, and EC on PUBEB and PRIEB. Next, mediation analysis is suitable for exploring the indirect effects between variables, helping to understand the mechanisms through which EF and EC mediate the relationship between CNE and PEB. Thus, mediation analysis is used to examine the mediating role of EF and EC between CNE and the two types of PEB, and to compare the magnitude of the mediating effects of these two variables. Moderation analysis can reveal how ERP influences the strength or direction of the relationship between other variables, thereby providing insights into the complex effects of ERP. Hence, moderation analysis is employed to analyze the moderating role of ERP in the relationships between EF, EC, and the two types of PEB. Finally, the moderating effect of ERP on the mediating effect is analyzed to further explore its role in the mediation pathways of EF and EC. The mathematical formulas constructed for the mediation and moderation analyses are shown in Equations 1–3.


(1) $$Y=c_{0}+c_{1}X+e_{1}$$

(2) $$M=a_{0}+a_{1}X+e_{2}$$

(3) $$Y=b_{0}+b_{1}X+b_{2}M+b_{3}W+b_{4}MW+e_{3}$$

In Equation 1, c1 represents the impact coefficient of the independent variable (childhood natural experience) on PEB. In Equation 2, a1 represents the impact coefficient of the independent variable on the mediating variable (EF or EC). In model (3), b1 represents the direct impact coefficient of childhood natural experience on PEB, b2 represents the impact coefficient of the mediating variable on PEB, b3 represents the impact coefficient of the moderating variable (ERP) on PEB, and b4 represents the impact of the interaction term of the mediating variable and the moderating variable on PEB.

## 4 Result

### 4.1 Correlation analysis among variables

To gain an initial understanding of the relationships between the variables, the correlations among the key variables were first analyzed. Table 2 presents the correlation matrix among the core variables. The correlation between PUBEB and PRIEB, and ERP is not significant. However, it has a strong correlation with EF (*r* = 0.315, *p* < 0.001). PRIEB has a significant correlation with ERP, EC, EF, and childhood natural experience. These four variables - ERP, EC, EF, and childhood natural experience - all have significant correlations with each other. The correlation matrix in Table 2 indicates that there is a certain correlation among the main core variables of this paper, but the correlation coefficient between each variable is less than 0.7. Therefore, the risk of multicollinearity in subsequent regression analysis is low (Mela and Kopalle, 2002; Dormann et al., 2013).

**Table 2:** Core variable correlation matrix.

	PUBEB	PRIEB	ERP	EC	EF	CNE
Public sphere pro-environmental behavior (PUBEB)	1.000					
Private sphere pro-environmental behavior (PRIEB)	0.000	1.000				
Environmental risk perception (ERP)	0.002	0.269^***^	1.000			
Environmental contacts (EC)	0.144^***^	0.248^***^	0.081^**^	1.000		
Environmental feelings (EF)	0.315^***^	0.223^***^	0.219^***^	0.187^***^	1.000	
Childhood nature experiences (CNE)	0.073^**^	0.100^***^	0.051^*^	0.143^***^	0.297^***^	1.000

^*^p < 0.05, ^**^p < 0.01, ^***^p < 0.001.

### 4.2 Influential factors of pro-environmental behavior

To effectively analyze the strength and direction of the influence of independent variables, mediating variables, and moderating variables on the dependent variable, this study employs a multiple linear regression method to examine the effects of CNE, EF, EC, and ERP on PEB. This paper categorizes PEB into PUBEB and PRIEB. Table 3 separately analyses the impacts on PUBEB and PRIEB.

**Table 3:** Regression analysis of factors influencing public and private sphere environmental behavior.

	Public-sphere environmental behavior(PUBEB)				Private-sphere environmental behavior (PRIEB)			
	Model 1	Model 2	Model 3	Model 4	Model 5	Model 6	Model 7	Model 8
Gender (Male = 1)	0.078	0.077	0.089^+^	0.076	−0.337^***^	−0.338^***^	−0.315^***^	−0.283^***^
	(0.050)	(0.050)	(0.048)	(0.048)	(0.051)	(0.051)	(0.049)	(0.048)
Age	−0.004	−0.004	−0.005^*^	−0.005^*^	0.016^***^	0.016^***^	0.013^***^	0.014^***^
	(0.002)	(0.002)	(0.002)	(0.002)	(0.003)	(0.003)	(0.002)	(0.002)
Place of residence (Urban = 1)	−0.210^***^	−0.202^***^	−0.187^***^	−0.180^***^	0.164^**^	0.178^**^	0.190^***^	0.171^**^
	(0.057)	(0.057)	(0.055)	(0.054)	(0.058)	(0.057)	(0.055)	(0.054)
Annual household income	0.005^**^	0.004^**^	0.004^**^	0.004^*^	−0.002	−0.002	−0.002	−0.001
	(0.002)	(0.002)	(0.001)	(0.001)	(0.002)	(0.002)	(0.001)	(0.001)
Educational level	0.065^***^	0.064^***^	0.047^***^	0.048^***^	0.035^***^	0.034^***^	0.017^+^	0.014^+^
	(0.009)	(0.009)	(0.009)	(0.009)	(0.009)	(0.009)	(0.009)	(0.009)
Childhood nature experiences (CNE)		0.063^*^	−0.024	−0.026		0.108^***^	0.024	0.030
		(0.025)	(0.025)	(0.025)		(0.025)	(0.025)	(0.025)
Environmental feelings (EF)			0.241^***^	0.258^***^			0.199^***^	0.163^***^
			(0.026)	(0.026)			(0.026)	(0.026)
Environmental contacts (EC)			0.114^***^	0.115^***^			0.175^***^	0.165^***^
			(0.025)	(0.025)			(0.025)	(0.024)
Environmental risk perception (ERP)				−0.089^**^				0.210^***^
				(0.025)				(0.025)
EF × ERP				0.017				0.055^*^
				(0.023)				(0.023)
EC × ERP				0.047^+^				−0.055^*^
				(0.025)				(0.025)
Constant	−0.625^**^	−0.628^**^	−0.370^*^	−0.376^*^	−0.969^**^	−0.973^**^	−0.688^**^	−0.688^**^
	(0.183)	(0.183)	(0.177)	(0.177)	(0.186)	(0.185)	(0.179)	(0.175)
*N*	1,499	1,499	1,499	1,499	1,499	1,499	1,499	1,499
Adjusted *R^2^*	0.087	0.090	0.159	0.167	0.057	0.068	0.140	0.187
*F*	29.475	25.739	36.487	28.333	19.023	19.164	31.523	32.287
*p*	0.000	0.000	0.000	0.000	0.000	0.000	0.000	0.000

Standard errors in parentheses; ^+^*p* < 0.1, ^*^p < 0.05, ^**^p < 0.01, ^***^p < 0.001.

In Table 3, we constructed eight models. The dependent variable in models 1 to 4 is PUBEB. Model 1 serves as the baseline model, which only analyzes the influence of control variables on PUBEB. Model 2, built on the basis of model 1, incorporates CNE. The analysis results indicate that CNE have a significant positive impact on PUBEB (*b* = 0.063, *p* < 0.1). Model 3, built on the basis of model 2, introduces EF and EC. Statistical results show that both variables have a significant positive impact on PUBEB (EF: *b* = 0.241, *p* < 0.001; EC: *b* = 0.114, *p* < 0.001). Model 4 introduces ERP and its interaction with EF and EC. Statistical results indicate that ERP has a significant negative impact on PUBEB (*b* = −0.089, *p* < 0.01). The regulatory effect of ERP on the relationship between EF and PUBEB is not significant (*b* = 0.017, *p* > 0.1). The regulatory effect of ERP on the relationship between EC and PUBEB is significant (*b* = 0.047, *p* < 0.1).

The dependent variable in models 5 to 8 is PRIEB. Model 5 serves as the baseline model, which only examines the influence of control variables on PRIEB. Model 6, built on the basis of model 5, incorporates CNE. These experiences significantly impact PRIEB (*b* = 0.108, *p* < 0.001). Model 7 built on the basis of model 6, introduces EF and EC. Statistical results show that both variables have a significant positive impact on PRIEB (EF: *b* = 0.199, *p* < 0.001; EC: *b* = 0.175, *p* < 0.001). Model 8 introduces ERP and its interaction with EF and EC. Statistical results indicate that ERP has a significant positive impact on PRIEB (*b* = 0.210, *p* < 0.001). The positive regulation of the relationship between EF and PRIEB by ERP is significant (*b* = 0.055, *p* < 0.05). Although the negative regulatory effect of EC on the relationship with PRIEB is also significant (*b* = −0.055, *p* < 0.05).

### 4.3 Mediating effects of environmental feelings and environmental contacts

To further validate the mediating roles of EF and EC between childhood natural experiences and two types of PEB, this study employed the Bootstrap analysis method (Streukens and Leroi-Werelds, 2016; Wang et al., 2019), iterating 2000 times under a 95% confidence interval for mediation effect analysis. The fit indicators of the mediation analysis model showed that the chi-square/degree of freedom was 2.937, less than the critical value of 3; GFI was 0.991, AGFI was 0.977, CFI was 0.975, all greater than 0.9; RMSEA was 0.036, less than 0.08. All fit indicators in the model fully met the requirements of fit (Hu and Bentler, 1999), indicating good overall fit of the mediation analysis model.

As shown in Table 4, in PUBEB, the mediation effect value of EF were 0.077 (*p* < 0.01), and the mediation effect value of EC were 0.017 (*p* < 0.01). Subtracting the mediation effect of EC (b) from the mediation effect of EF (a), the difference (c) in their mediation effects was significant (*b* = 0.060, *p* < 0.01), indicating that the mediation effect of EF were greater than that of EC between childhood natural experience and PUBEB. In PRIEB, the mediation effect value of EF were 0.048 (*p* < 0.01), and the mediation effect value of EC were 0.024 (*p* < 0.01), both variables had significant mediation effect values. Subtracting the mediation effect of EC (e) from the mediation effect of EF (d), the difference (f) in their mediation effects was significant (*b* = 0.024, *p* < 0.05), indicating that the mediation effect of EF were greater than that of EC between childhood natural experience and PRIEB.

**Table 4:** Comparison and verification of the mediating effect of environmental feelings on environmental contacts.

Path name	Path	*b*	SE	*p*	Lower	Upper
a	CNE → NF → PUBEB	0.077	0.010	0.003	0.056	0.097
b	CNE → NC → PUBEB	0.017	0.005	0.001	0.009	0.027
c	a - b	0.060	0.011	0.003	0.036	0.081
d	CNE → NF → PUBEB	0.048	0.010	0.002	0.031	0.069
e	CNE → NC → PUBEB	0.024	0.005	0.002	0.014	0.036
f	d - e	0.024	0.012	0.031	0.002	0.047

CNE, Childhood nature experiences; EF, Environmental feelings; EC, Environmental contacts; PUBEB, Public-sphere pro-environmental behavior; PRIEB, Private-sphere pro-environmental behavior.

### 4.4 The moderating effect of environmental risk perception

**Figure 2:**
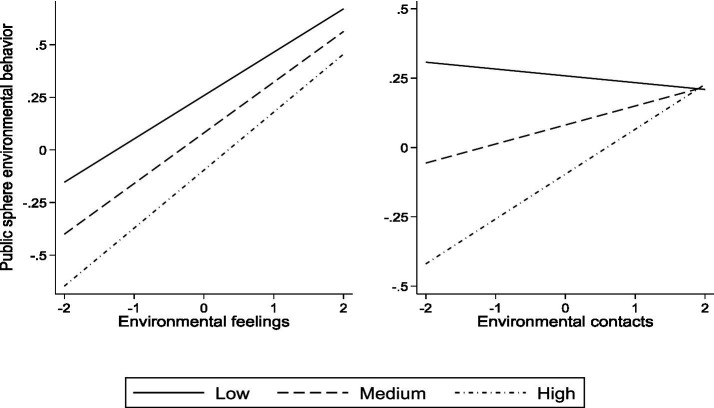
Moderation diagram of environmental risk perception on the relationship between environmental feelings, environmental contacts, and public sphere pro-environmental behavior.

**Figure 3:**
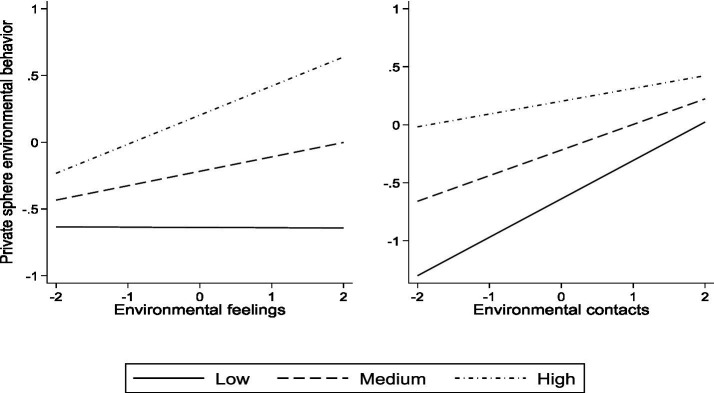
Moderation diagram of environmental risk perception on the relationship between environmental feelings, environmental contacts, and private sphere pro-environmental behavior.

Models 4 and 8 in Table 3 have already presented the moderating effects of ERP on the relationships between EF, EC, and two types of PEB. To more intuitively present the moderating effects, this paper has separately created the moderating graphs of the relationships between EF, EC, and two types of PEB under different levels of ERP, as shown in Figures 2, 3.

Figure 2 presents two graphs illustrating the moderating effects of ERP. The left graph depicts the relationship between ERP, EF, and PUBEB. The right graph, on the other hand, shows the relationship between ERP, EC, and PUBEB. As shown in Figure 2, residents with low ERP generally exhibit a higher level of PUBEB compared to those with medium and high levels of ERP. As the level of EF increases, there are no significant intersection points in PUBEB among residents with different levels of ERP, indicating that ERP does not significantly moderate the relationship between EF and PUBEB. However, ERP does have a significant positive moderating effect on the relationship between EC and PUBEB. This is evidenced by the clear intersection points in PUBEB among residents with different levels of ERP as their level of EC increases. When residents’ ERP is low, EC appears to negatively impact PUBEB. However, as residents’ ERP increases, the slope of EC on PUBEB becomes steeper, indicating a stronger positive impact.

Figure 3 presents two moderating graphs. On the left, the graph illustrates the relationship between ERP, EF, and PRIEB. On the right, the graph depicts the relationship between ERP, EC, and PRIEB. As demonstrated in Figure 3, when residents’ ERP is high, the overall level of residents’ PRIEB is higher compared to those with medium and low levels of ERP. Additionally, the impact of EF on PRIEB varies among residents with different levels of ERP, resulting in distinct slopes. Notably, as the level of EF decreases, the three lines intersect. Furthermore, the positive moderating effect of ERP on the relationship between EF and PRIEB becomes more pronounced as residents’ ERP increases. Conversely, the relationship between EC and PRIEB exhibits significant negative moderation. Residents with varying levels of ERP show distinct slopes for the impact of EC on PRIEB. When the value of EC is high, the three lines also intersect. Specifically, residents with higher ERP experience a smaller impact of EC on their PRIEB compared to those with lower ERP.

### 4.5 Moderation of environmental risk perception on mediation effects

To examine the moderating effect of ERP on the mediation of EF and EC, we employed the mediation moderation test proposed by Preacher and colleagues in 2007 (Preacher and Hayes, 2008). Additionally, we utilized Bootstrap analysis to calculate the mediation effects at different levels of ERP, with 2000 iterations and a 95% confidence interval. The results, as summarized in Table 5, demonstrate that varying levels of ERP led to distinct mediation effects for both EF and EC.

**Table 5:** Moderated mediation analysis.

Mediation path	Moderator value	*b*	SE	*Z*	*p*	Lower	Upper
CNE → EF → PUBEB	Low	0.095	0.013	7.202	0.000	0.069	0.120
	Medium	0.101	0.012	8.753	0.000	0.078	0.124
	High	0.107	0.015	7.263	0.000	0.078	0.136
CNE → EC → PUBEB	Low	0.011	0.006	1.775	0.076	−0.001	0.023
	Medium	0.019	0.005	3.663	0.000	0.009	0.030
	High	0.027	0.007	3.929	0.000	0.014	0.041
CNE → EF → PRIEB	Low	0.032	0.017	1.984	0.048	0.000	0.065
	Medium	0.049	0.010	5.232	0.000	0.031	0.067
	High	0.066	0.013	5.123	0.000	0.040	0.091
CNE → EC → PRIEB	Low	0.037	0.009	4.264	0.000	0.020	0.054
	Medium	0.032	0.007	4.686	0.000	0.018	0.045
	High	0.027	0.007	3.981	0.000	0.014	0.040

CNE, Childhood nature experiences; EF, Environmental feelings; EC, Environmental contacts; PUBEB, Public sphere pro-environmental behavior; PRIEB, Private sphere pro-environmental behavior.

In the context of PUBEB, the mediation effect of EF and EC varies across different levels of ERP. Specifically, as residents’ perception of environmental risk intensifies, the mediation effect of EF and EC between CNE and PUBEB also strengthens. That is, ERP, acting as a moderating variable, has a positive regulatory effect on the mediation effect of EF and EC in the relationship between CNE and PUBEB.

Conversely, in the context of PRIEB, the mediation effect of EF and EC also exhibits differences based on varying levels of ERP. As residents’ perception of environmental risk increases, the mediation effect of EF between CNE and PRIEB becomes more pronounced, while the mediation effect of EC between CNE and PRIEB diminishes.

## 5 Discussion

### 5.1 The discussion of the results

This study, under the theoretical framework of the pro-life hypothesis, innovatively reveals the mechanisms through which CNE influence adult PEB, with a particular focus on analyzing the mediating effects of EF and EC. Additionally, it provides an in-depth analysis of the moderating role of ERP. The study presents two key innovations: first, it clearly distinguishes and compares the lasting impact of EF with the contextual effects of EC; second, it examines in detail how the mediating effects of EF and EC vary under different levels of ERP, offering valuable insights for the precise design of strategies to engage residents in environmental protection actions. The findings of this study indicate that CNE primarily exert an indirect influence on PEB through EF and EC. ERP has varying moderating effects on the relationships between EF, EC, and the two types of PEB. Moreover, ERP significantly moderates the mediating effects of EF and EC. The following sections discuss these findings in detail.

While some previous studies have explored the impact of CNE on adulthood PEB, few have conducted detailed analyses of the underlying mechanisms (Rosa and Collado, 2019; Chawla, 2021). Our study astutely recognizes the need for innovative research in this area. Contrary to prior findings, we reveal that CNE do not directly influence adulthood PEB. Instead, they operate indirectly through EF and contacts during adulthood. This underscores that CNE represent early sensory impressions from nature, which, after prolonged attenuation, do not directly drive adulthood behavior. We offer two explanatory perspectives. First, the combined view of feelings activation theory and feelings functionality theory emphasizes the pivotal role of EF. During childhood, interactions with the natural environment evoke diverse feelings, serving as both the foundation for emotional functioning and the basis for establishing connections with the natural world. However, the critical question lies in whether these childhood-formed EF can be effectively transmitted and continuously activated during adulthood. The persistence of an individual’s feeling connection and cognition toward the environment largely depends on their childhood interactions and feeling cultivation with nature. If frequent childhood contacts to nature fosters deep emotional attachment, it becomes more readily rekindled during adulthood, thereby motivating support for environmental protection and sustainable development. Conversely, a lack of feeling attachment to nature during childhood or insufficient ongoing interaction with nature during adulthood may lead to environmental indifference, subsequently affecting an individual’s level of engagement in environmental protection. Therefore, CNE play a decisive role in shaping adult environmental awareness and behavior. The second perspective is the explanation from the viewpoint of natural connection. The theory of natural connection emphasizes the importance of an individual’s contact with the natural environment, asserting that such contact allows individuals to experience the charm and interest of nature(Cleary et al., 2017). In the process of interacting with nature, individuals not only experience the direct pleasure brought by natural scenery, but also develop a temporary attachment and protective behavior toward the natural environment driven by the context. This specific context often prompts people to take environmental protection actions in the present, such as picking up trash and rescuing small animals. Although these context-driven environmental behaviors may be impromptu, their positive impact cannot be ignored. In the process of contact with the natural environment, the sense of participation integrated into the natural environment is also important for individuals to form a protective consciousness. This process of natural participation strengthens individuals’ sense of responsibility for the environment, stimulates their environmental actions, and makes environmental protection a conscious behavior.

The most significant finding of this study is that in the transition from childhood natural experiences to adulthood PEB, the enduring impact of EF surpasses the contextual stimulation effect of EC. Stable and persistent EF refer to an individual’s long-term positive or negative feelings responses to the environment, typically stemming from prolonged interaction and deep emotional connections with the surroundings. These stable and persistent environmental emotions can foster a sense of responsibility and belonging, manifesting as intrinsic emotional motivation for PEB. While brief EC may temporarily stimulate an individual’s interest in the environment and evoke positive reactions within specific contexts, once removed from those specific natural environments, the individual’s interest and attention toward nature tend to diminish, making sustained environmentally friendly actions challenging. The Dual-Factor Theory of Emotion-Behavior posits that stable and enduring environmental emotions serve as intrinsic motivation. Individuals inherently imbue themselves with a sense of environmental responsibility, which can continuously drive them to adopt environmentally friendly behaviors, rather than merely being stimulated by external factors in specific situations (Jin et al., 2020). Although prolonged contact to the environment can also generate EF and a sense of environmental responsibility in individuals, the transient nature of context-specific environmental contact does not ensure the continuity of environmental commitment. Therefore, greater emphasis should be placed on nurturing EF during childhood natural experiences, extending environmental care sentiments into adulthood, and complementing these efforts with positive environmental encounters. Only when EF and EC work in tandem can they effectively drive adult PEB.

This study reveals the “dual moderation differentiation” in ERP on the relationships between EF, EC, and two types of PEB. Specifically, ERP exhibits dual differentiation in its role in stimulating the positive effects of EF and EC on two types of PEB. First, the moderating effect of ERP on the relationship between EF and PEB is differentiated. According to the affect-behavior theory, when EF provide strong intrinsic motivation for PEB, situational factors are not required to directly promote the behavior (Böhm and Pfister, 2008); conversely, when the intrinsic motivation of EF is weaker, situational factors become an additional driving force for behavior formation. In the case of PUBEB, individuals with stronger EF are likely to take action regardless of their level of ERP, thereby masking the moderating effect of ERP. However, the effect of EF on PRIEB is not as strong as it is on PUBEB. The “threat explicitness” of ERP opens up the cognitive-practical channel for the transformation of EF into PRIEB, resulting in a significant moderating effect of ERP on the relationship between EF and PRIEB. Second, ERP also shows distinct moderation in the relationship between EC and pro-environmental behaviors. In public behaviors, low ERP de-responsibilizes EC, meaning individuals view nature experiences as leisure activities rather than public responsibility, which negatively impacts the relationship between EC and public behaviors. Conversely, high ERP, through a risk framing process, redefines EC as a tool for reinforcing environmental cognition, significantly enhancing PUBEB (Zhang et al., 2024a). In PRIEB, ERP triggers residents to form varying degrees of environmental responsibility, encouraging EC to enhance PRIEB. However, as perceived environmental risk increases, individuals are constrained by the difficulty of problem-solving, and situational EC alone is insufficient to quickly increase PRIEB. As a result, the effect of EC on PRIEB exhibits diminishing marginal returns. The moderating effect of ERP on the relationship between EC and PUBEB is more positive. The phenomenon of “dual moderation differentiation” in ERP indicates that ERP is more effective in stimulating the enhancement of EF in relation to PRIEB, while it more effectively stimulates the enhancement of EC in relation to PUBEB.

The present study also discovered that ERP plays a regulatory role in the mediation effect of EF and EC between CNE and adulthood PEB. In the context of China’s modernization process, which involves urbanization and industrialization, environmental pollution varies across different regions. Residents’ perception of environmental risk tends to strengthen the impact of EF and contacts on PEB. Specifically, as perceived environmental risk intensifies, the influence of EF and contacts on behavior becomes more pronounced, and the mediation effect of EF and contacts strengthens. However, the mediation effect of EC between CNE and adult PRIEB diminishes as perceived environmental risk increases. Three potential explanations are proposed. First, heightened ERP may directly stimulate PRIEB. As individuals become more aware of potential environmental threats, they may feel the need to take immediate measures to protect their environmental safety. This proactive approach could reduce reliance on EC as a catalyst for behavior. Essentially, increased urgency and self-protective awareness may lead to direct responses to environmental risk without necessarily relying on the gradual influence typically provided by EC. Second, the quality and frequency of EC may be adversely affected by heightened risk perception. In environments with elevated ERP, individuals may instinctively limit their interactions with the natural environment. This could be due to concerns about personal safety or avoidance of potential hazards. Reduced quantity and quality of environmental exposure may weaken the driving force of EC for PRIEB, thus diminishing its mediation effect. Third, increased ERP may activate other psychological mechanisms that influence PRIEB. Beyond the impact of EC on PRIEB, risk perception itself may trigger a range of psychological responses, such as risk avoidance and self-protection. These mechanisms can alter individuals’ perceptions of their efficacy in performing PRIEB. If an individual perceives that broader environmental risks are beyond their control, reducing their sense of efficacy in engaging in PRIEB (Yan et al., 2024), this shift in mindset may decrease the motivating effect of EC on such behavior.

### 5.2 The theoretical implications

This article makes a certain theoretical contribution. This study, based on the framework of the Biophilia Hypothesis, provides an in-depth analysis of how CNE profoundly influence PEB in adulthood through the dual pathways of the stability and durability of EF and situational EC. The Biophilia Hypothesis emphasizes the close relationship between an individual’s environmental behavior and their feelings connection to nature (Wilson, 1984). Especially during early childhood, these feeling experiences continue to affect an individual’s PEB in adulthood through various mechanisms (Rosa and Collado, 2019). The core contribution of this study lies in systematically revealing the mediating role of EF and EC in the relationship between CNE and PEB in adulthood. Specifically, EF play a crucial role in the formation of PEB, particularly in promoting long-term sustainable behaviors, further validating the importance of EF as an intrinsic motivator. This finding extends the understanding of emotional motivation in PEB research and provides empirical support for related theories. Additionally, this study found that situational EC have a significant short-term stimulating effect on an individual’s PEB, indicating that the generation of PEB is not only reliant on emotional motivation but is also strongly influenced by situational factors (Gifford, 2007). This result supports the context-dependent PEB model and offers a new understanding of the mechanisms underlying immediate behavioral responses. Therefore, the theoretical framework based on the perspective of sensory practices—analyzing CNE from the standpoint of individual emotions and perceptions—offers a novel viewpoint for explaining the temporal and spatial formation of PEB. Through this perspective, this study not only constructs a multi-level theoretical model for the formation of PEB but also provides innovative theoretical pathways for the deepening and expansion of sensory PEB theory, further enriching the existing theoretical system and advancing its application and expansion across different contexts (Chang et al., 2019).

### 5.3 The managerial implications

This article also has certain managerial implications. This study emphasizes the importance of outdoor nature experiences in nurturing children’s EF. When children engage more directly with the natural environment, it fosters a genuine love and care for nature. EF, being stable and enduring, can continue to drive individuals to engage in PEB in adulthood. Therefore, to encourage greater participation in environmental protection, it is essential to enhance children’s interaction with the natural environment and reinforce their emotional connection to nature through such contacts. Furthermore, this study suggests that EF should be cultivated from childhood, using nature experiences to develop the next generation’s sense of environmental responsibility and action. This implies that families, schools, and communities should collaborate to provide children with rich nature experiences, enabling them to establish positive connections with nature through play, exploration, and learning. Both unstructured natures contact and planned, educational outdoor activities are equally important, not only laying a solid foundation for children’s overall development but also fostering more active participants and advocates for future environmental conservation efforts.

### 5.4 The limitations and future research avenues

This article also has several limitations. First, there are limitations in measuring CNE. In this study, we relied on participants’ recollections to assess their nature experiences during childhood. Given that these experiences occurred in childhood, alternative methods of measurement are difficult to implement. However, participants’ inexact memories may introduce measurement errors. Future research should prioritize longitudinal studies to refine measurement techniques and minimize these errors. Second, there are limitations in the assumptions about the relationships between variables. This study primarily focuses on comparing the mediating effects of EF and EC between CNE and adulthood environmental behaviors. As a result, it does not examine the impact of EC on EF, but this remains a topic worthy of exploration. Subsequent research could focus on investigating the relationship between EC and EF, further elucidating the mechanisms through which these factors influence environmental behaviors. Future research could employ qualitative methodologies to examine the differential impacts and underlying mechanisms through which nature experiences facilitated by diverse demographic groups during childhood shape pro-environmental behaviors in adulthood.

## 6 Conclusion

This study reveals the mechanisms through which CNE influence PEB in adulthood. First, CNE have no direct effect on PEB in adulthood, indicating that relying solely on childhood EC does not directly shape adult PEB. Second, CNE exert their indirect effects primarily through EF and EC, with the mediating effect of EF being stronger than that of EC, highlighting the critical role of emotions in driving PEB. Finally, ERP significantly moderates the relationship between EC and PEB, as well as the connection between EF and private PEB, thereby strengthening the mediating effects of both EF and EC. These findings emphasize the equal importance of unstructured natural contact and planned educational outdoor activities during childhood. Society should provide children with abundant opportunities for nature experiences, nurture EF, and foster a close connection with nature, laying a foundation for cultivating future environmental conservation participants and advocates.

## Data Availability

The raw data supporting the conclusions of this article will be made available by the authors, without undue reservation.
