# Supplementary material for: Driven by feelings or stimulated by context: how childhood nature experience shaped adulthood pro-environmental behavior?
Source: Front Psychol. 2025 Mar 3;16:1529388. doi: 10.3389/fpsyg.2025.1529388 (PMC11911382; doi:10.3389/fpsyg.2025.1529388)
Supplement: Supplementary file 1 [file Table_1.docx]

**Survey Questions (English)**

**1. What is your gender?**

1) Male 2) Female

**2. What is your date of birth? Year Month**

**3. In the past year, which type of area do you and your family usually reside in?**

1) Village 2) Town 3) County town 4) Suburbs of a city above the county level

5) Urban area of a city above the county level

**4. What was your household's total annual income (after tax) last year? Ten thousand yuan.**

**5. What is your highest level of education (including current studies)?**

1) Primary school or below 2) Junior high school 3) High school

5) Bachelor's degree 6) Graduate degree or above

**6. How often do you engage in the following activities or behaviors?**

|  | Never | Rarely | Sometimes | Often | Always |
| --- | --- | --- | --- | --- | --- |
| Growing green plants at home |  |  |  |  |  |
| Maintaining cleanliness around the house |  |  |  |  |  |
| Sorting waste for disposal |  |  |  |  |  |
| Discussing environmental issues with relatives and friends |  |  |  |  |  |
| Buying appliances and daily necessities with lower energy consumption and less environmental pollution |  |  |  |  |  |
| Willing to save water due to the decrease in global freshwater resources |  |  |  |  |  |
| Turning off the lights when leaving the room as the last person |  |  |  |  |  |
| Bringing your own shopping basket or bag when purchasing daily items |  |  |  |  |  |
| Reusing plastic packaging bags |  |  |  |  |  |
| Buying fruits and vegetables that have not been treated with chemical fertilizers and pesticides |  |  |  |  |  |
| Donating for the environmental protection of the Yangtze or Yellow River basin |  |  |  |  |  |
| Actively paying attention to environmental issues and information reported in radio, television, and newspapers |  |  |  |  |  |
| Participating in environmental propaganda organized by the village committee (or residents' committee) |  |  |  |  |  |
| Participating in environmental sanitation public welfare activities organized by the workplace |  |  |  |  |  |
| Participating in environmental activities organized by non-governmental environmental groups |  |  |  |  |  |
| Paying for the maintenance of forests or green spaces |  |  |  |  |  |
| Actively participating in complaints and appeals for the resolution of environmental issues |  |  |  |  |  |

**7. How much do the following statements match your actual experiences during your childhood?**

|  | Strongly Agree | Agree | Neutral | Disagree | Strongly Disagree |
| --- | --- | --- | --- | --- | --- |
| I participated in many interesting outdoor activities when I was young. |  |  |  |  |  |
| I always found joy in the gardens or fields near my home when I was young. |  |  |  |  |  |
| I felt very happy when I went to the forest with my parents during my childhood. |  |  |  |  |  |
| The streams and fields of my hometown during my childhood always brought me joy. |  |  |  |  |  |

**8. Below are some statements. Please select your attitude towards each one.**

|  | Strongly Agree | Agree | Neutral | Disagree | Strongly Disagree |
| --- | --- | --- | --- | --- | --- |
| I am inseparable from nature; I am part of it. |  |  |  |  |  |
| No matter where I am, I intentionally pay attention to local wildlife. |  |  |  |  |  |
| I feel closely connected to the Earth and all living beings. |  |  |  |  |  |
| Even in the city center, I pay attention to the surrounding natural environment. |  |  |  |  |  |
| I enjoy being outdoors even when the weather is not good. |  |  |  |  |  |
| I always consider the impact of my actions on the environment. |  |  |  |  |  |

**9. In the past six months, how often have you tended to your indoor plants or flowers (watering, loosening soil, fertilizing, pruning leaves, etc.)?**

(1) Never (2) 1-2 times per month (3) Once a week (4) Every 2-3 days (5) Almost every day

**10. In the past six months, how often have you been in contact with natural environments (visiting parks, walking in neighborhood green spaces, going on outings, hiking, etc.)?**

(1) Never (2) 1-2 times per month (3) Once a week (4) Every 2-3 days (5) Almost every day

**11. In the past six months, how often have you participated in outdoor activities (such as hiking, cycling, camping)?**

(1) Never (2) 1-2 times per month (3) Once a week (4) Every 2-3 days (5) Almost every day

**13. How serious do you think the following environmental issues or risks are to us?**

|  | Not Serious | Somewhat Serious | Neutral | Serious | Very Serious |
| --- | --- | --- | --- | --- | --- |
| Air pollution |  |  |  |  |  |
| Water pollution |  |  |  |  |  |
| Noise pollution |  |  |  |  |  |
| Soil pollution |  |  |  |  |  |
| Household waste pollution |  |  |  |  |  |
| Industrial waste pollution |  |  |  |  |  |
| Deforestation |  |  |  |  |  |
| Degradation of arable land quality |  |  |  |  |  |
| Reduction of wild flora and fauna |  |  |  |  |  |
| Freshwater scarcity |  |  |  |  |  |
| Climate warming |  |  |  |  |  |
| Food pollution |  |  |  |  |  |
| Insufficient green spaces |  |  |  |  |  |

**调查问卷（中文）**

**1. 您的性别是？**

1）男 2）女

**2. 您的出生日期是：** 年 月

**3. 请问最近这一年，您与家人常居住的地区属于哪一类？**

1）村庄 2）乡镇所在地 3）县城 4）县城以上的城市的郊区 5）县城以上城市的市区

**4. 您的家庭去年（ 2019 ）全年的总收入（税后）是** **万元？**

**5. 您目前的最高教育程度是（包括目前在读的）**

1）小学及以下 2）初中 3）高中、中专、中师、技校

4）专科、高职、成人专科 5）本科、成人本科 6）研究生及以上

**6. 您从事下列活动或行为的情况的频率如何？**

|  | 完全不会 | 较少 | 一般 | 较多 | 经常 |
| --- | --- | --- | --- | --- | --- |
| 在家种植绿色植物 |  |  |  |  |  |
| 做好房屋周边环境卫生 |  |  |  |  |  |
| 对垃圾进行分类投放 |  |  |  |  |  |
| 与自己的亲戚朋友讨论环保问题 |  |  |  |  |  |
| 购买能耗更低、环境污染更小的家电和生活用品 |  |  |  |  |  |
| 全球淡水资源变少，我愿意为此节约用水 |  |  |  |  |  |
| 当我是最后一个离开房间时，我会随手关灯 |  |  |  |  |  |
| 采购日常用品时自己带购物篮或购物袋 |  |  |  |  |  |
| 对塑料包装袋进行重复利用 |  |  |  |  |  |
| 购买没有施用过化肥和农药的水果和蔬菜 |  |  |  |  |  |
| 为长江或黄河流域的环境保护捐款 |  |  |  |  |  |
| 主动关注广播、电视和报刊中报道的环境问题和环保信息 |  |  |  |  |  |
| 参加村委会（或居委会）组织的环境宣传 |  |  |  |  |  |
| 参加工作单位组织的环境卫生公益活动 |  |  |  |  |  |
| 参加民间环保团体举办的环保活动 |  |  |  |  |  |
| 自费养护树林或绿地 |  |  |  |  |  |
| 积极参加要求解决环境问题的投诉、上诉 |  |  |  |  |  |

**7. 下面的说法与您童年时期的实际情况是否一致？**

|  | 非常一致 | 比较一致 | 一般 | 不太一致 | 完全不一致 |
| --- | --- | --- | --- | --- | --- |
| 小时候我参加了很多有趣的户外活动 |  |  |  |  |  |
| 我小时候总能在家附近的花园或农田里找到乐趣 |  |  |  |  |  |
| 童年时我与父母到森林里去时，我感到非常幸福 |  |  |  |  |  |
| 童年家乡的溪边和田野总能给我带来快乐 |  |  |  |  |  |

**8** **下面列出了一些说法，请分别选择您的态度。**

|  | 完全赞同 | 比较赞同 | 说不清 | 不赞同 | 完全不赞同 |
| --- | --- | --- | --- | --- | --- |
| 我与自然不可分离，我属于自然的一部分 |  |  |  |  |  |
| 无论我身在何处，我都会有意去关注当地的野生动物 |  |  |  |  |  |
| 我觉得我与地球和所有生物都有密切联系 |  |  |  |  |  |
| 即使置身于城市中心，我也会注意周围的自然环境 |  |  |  |  |  |
| 即使天气不好，我也喜欢待在室外 |  |  |  |  |  |
| 我总是思考我的行为会对环境造成什么影响 |  |  |  |  |  |

**9. 您最近半年内，打理家里的植物或花草（浇水、松土、施肥、剪叶等）的频率大致是多久？**

（1）从未有过 （2）每月1—2次 （3）每周1次 （4）每隔2—3天 （5）几乎每天

**10. 您最近半年内，接触自然环境（逛公园、逛小区绿化带、郊游、爬山等）的频率大致是多久?**

（1）从未有过 （2）每月1—2次 （3）每周1次 （4）每隔2—3天 （5）几乎每天

**11. 您最近半年内，参与户外活动（如徒步、骑行、露营）的频率大致是多久?**

（1）从未有过 （2）每月1—2次 （3）每周1次 （4）每隔2—3天 （5）几乎每天

**12. 您认为下面的环境问题或风险对我们的危害大不大？**

|  | 不严重 | 不太严重 | 一般（说不清） | 比较严重 | 很严重 |
| --- | --- | --- | --- | --- | --- |
| 1.空气污染 |  |  |  |  |  |
| 2.水污染 |  |  |  |  |  |
| 3.噪音污染 |  |  |  |  |  |
| 4.土壤污染 |  |  |  |  |  |
| 5.生活垃圾污染 |  |  |  |  |  |
| 6.工业垃圾污染 |  |  |  |  |  |
| 7.森林植被破坏 |  |  |  |  |  |
| 8.耕地质量退化 |  |  |  |  |  |
| 9.野生动植物减少 |  |  |  |  |  |
| 10.淡水资源短缺 |  |  |  |  |  |
| 11.气候变暖 |  |  |  |  |  |
| 12.食品污染 |  |  |  |  |  |
| 13.绿地不足 |  |  |  |  |  |
